# P-62. Invasive Group A Streptococcus Infections in New Jersey: A Multicenter Review

**DOI:** 10.1093/ofid/ofaf695.291

**Published:** 2026-01-11

**Authors:** Brooke Schaeffer, Erica Lupiano, Liz Ricciardi, Eileen Yaney, Uzma Hasan, Robert Deutsch

**Affiliations:** Rowan University School of Osteopathic Medicine, Stratford, NJ; Rutgers Robert Wood Johnson Medical School, New Brunswick, New Jersey; Cooperman Barnabas Medical Center, Livingston, New Jersey; Cooperman Barnabas Medical Center, Livingston, New Jersey; Saint Barnabas Medical Center, West orange, New Jersey; Cooperman Barnabas Medical center, Livingston, NewJersey

## Abstract

**Background:**

Invasive Group A Streptococcus infections are on the rise at both the United States and international levels. Recent strains continue to progress to invasive disease with high rates of morbidity and mortality despite early diagnosis and appropriate treatment. This increase post-COVID pandemic may be related to altered immunity from lack of exposure, rising antimicrobial resistance, increased prevalence of risk factors like obesity, diabetes, and IV drug use, or the “triple-demic” with COVID, Influenza, and RSV spiking at the same time each year. All of these factors breed potential for invasive spread and outbreaks.

**Table 1:**
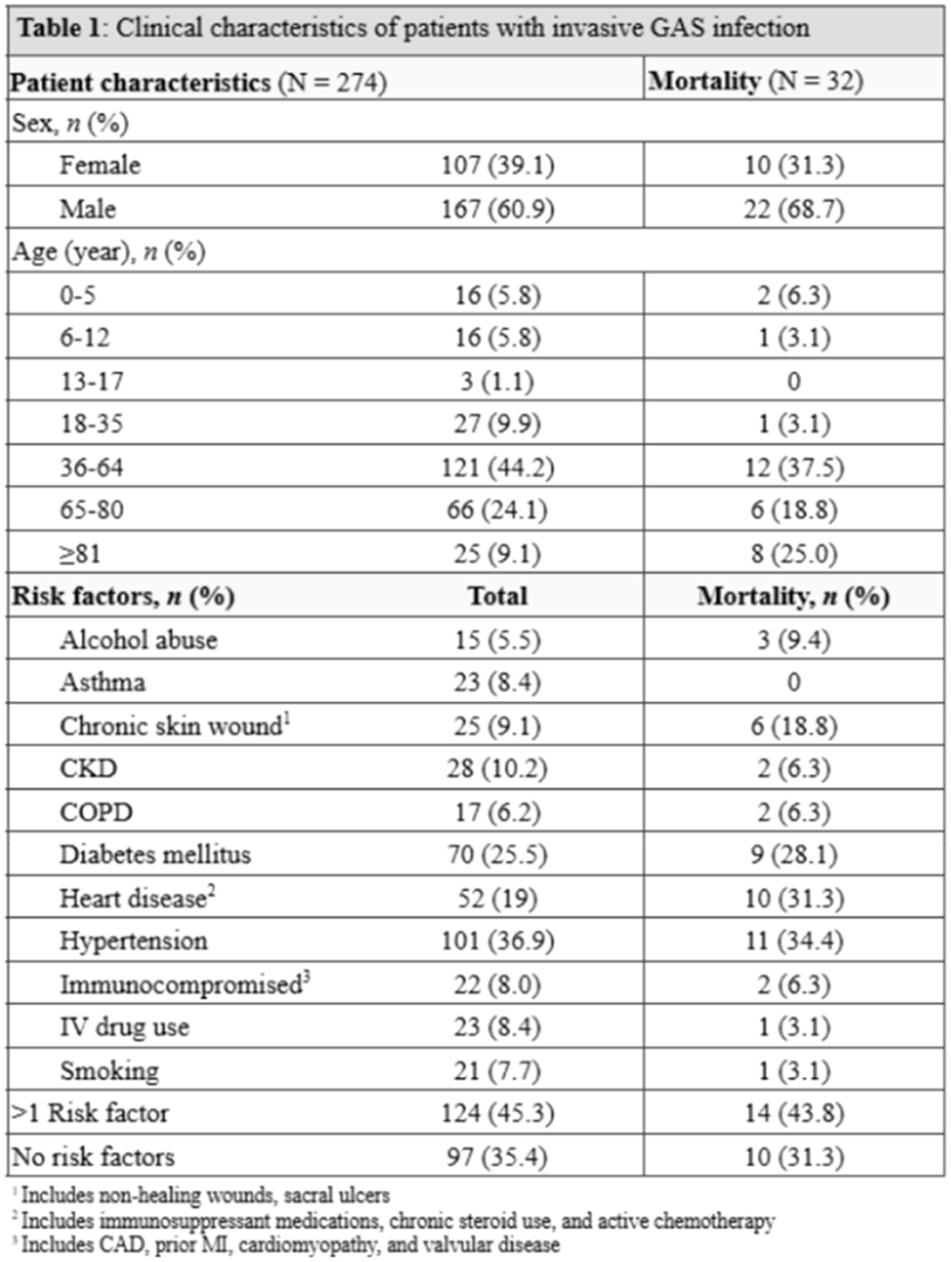
Clinical characteristics of patients with invasive GAS infections

**Figure 1:**
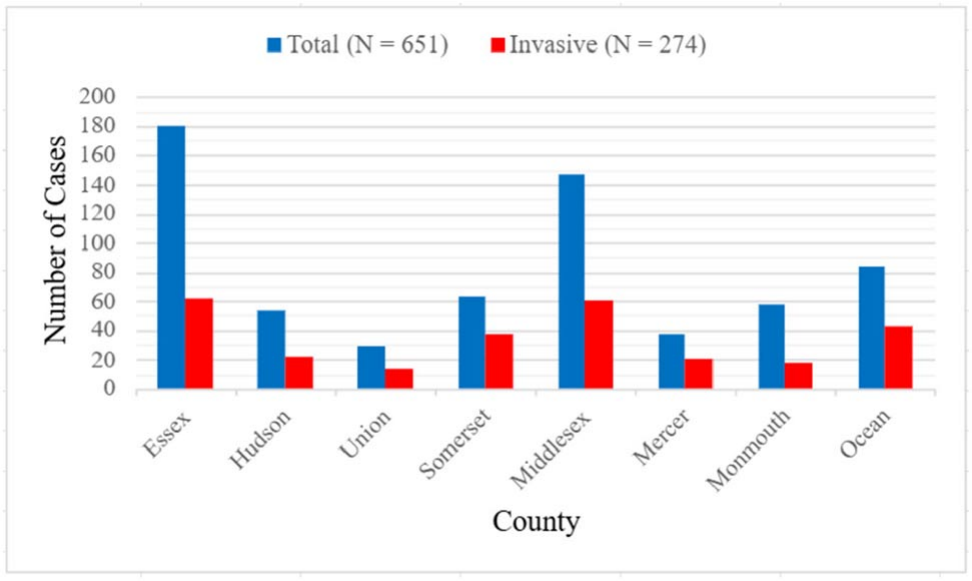
Invasive Cases in New Jersey by County (North to South)

**Methods:**

This is a retrospective review of 35 pediatric and 239 adult confirmed invasive Group A Strep infections from January 2022 through December 2024 across 12 Emergency Departments within a single healthcare system in New Jersey, USA. Invasivity was defined in accordance with the Centers for Disease Control Emerging Infections program. Descriptive analysis was performed for clinical presentations, epidemiology, morbidity, mortality, and antimicrobial susceptibility.

**Table 2:**
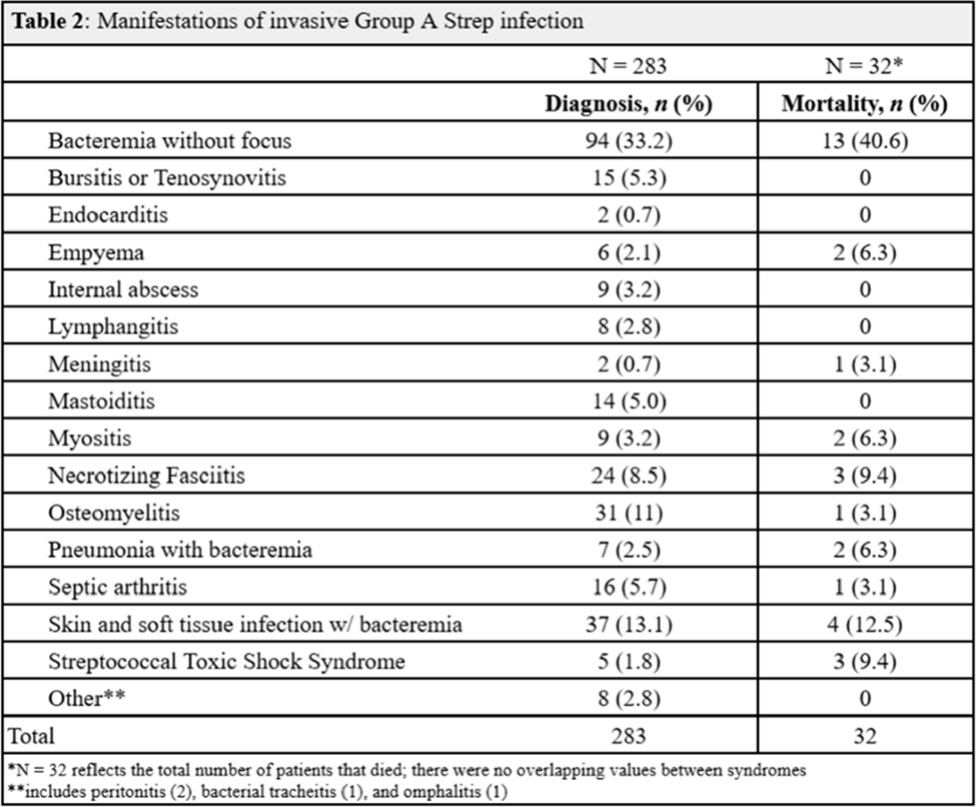
Manifestations of invasive Group A Strep infection

**Results:**

The mortality rate was 11.8% (32/274 patients); 29 adults and 3 children. The most frequently identified risk factors in adults were diabetes, chronic skin lesions, IV drug use, and having >1 underlying condition. Most pediatric patients did not have any underlying conditions. The most common presentations were bacteremia without focus (33.2%), skin and soft tissue infections associated with bacteremia (13.1%), and osteomyelitis (11%). Highest rates of infection were recorded in Somerset County (58.7%). Two cases of macrolide resistance and one case of penicillin resistance were observed.

**Conclusion:**

There should be a high clinical suspicion for invasive disease in older adults with history of hypertension, heart disease, or diabetes and current or recent Group A Strep infection. Children with no underlying medical conditions should still be considered at risk. Clinicians should consider cultures with susceptibility testing and track antibiotic resistance. Penicillin remains the optimal treatment.

**Disclosures:**

All Authors: No reported disclosures

